# Clinical outcomes of persistent sepsis-associated acute kidney injury in septic shock: a post-hoc analysis of a multicenter prospective cohort study

**DOI:** 10.1016/j.aicoj.2026.100041

**Published:** 2026-02-27

**Authors:** Michihito Kyo, Yu Kawazoe, Takeshi Morimoto, Hitoshi Yamamura, Kyohei Miyamoto, Noriko Miyagawa, Yoshinori Ohta, Kazuya Kikutani, Shinichiro Ohshimo, Nobuyuki Hirohashi, Nobuaki Shime

**Affiliations:** a Department of Emergency and Critical Care Medicine, Graduate School of Biomedical and Health Sciences, Hiroshima University, Hiroshima, Japan; b Department of Radiation Disaster Medicine, Research Institute for Radiation Biology and Medicine, Hiroshima University, Hiroshima, Japan; c Department of Emergency and Critical Care Medicine, Sendai Medical Center, Sendai, Japan; d Department of Data Science, Hyogo Medical University, Nishinomiya, Japan; e Department of Emergency and Critical Care Medicine, Japan Community Health Care Organization, Osaka Minato Central Hospital, Osaka, Japan; f Department of Emergency and Critical Care Medicine, Wakayama Medical University, Wakayama, Japan; g Department of Emergency and Critical Care Medicine, National Hospital Organization Kyoto Medical Center, Kyoto, Japan

**Keywords:** Sepsis, Shock, Vasopressor, Acute kidney injury, Renal failure

## Abstract

**Background:**

Sepsis-associated acute kidney injury (SA-AKI) contributes to a large morbidity and mortality burden. While emerging evidence suggests that the trajectory of SA-AKI, such as persistent SA-AKI, is associated with clinical outcomes, their association in septic shock remains unclear.

**Methods:**

In this multicenter prospective study in 20 ICUs, we investigated the incidence, clinical impact, and risk factors of persistent SA-AK in patients with septic shock requiring high-dose norepinephrine. Persistent SA-AKI was defined as an episode of AKI by KDIGO criteria lasting for at least 48 h in sepsis defined by sepsis-3 criteria. We assessed the association of persistent SA-AKI with clinical outcomes using multivariable Cox proportional hazards and logistic regression models. We also investigated risk factors for persistent SA-AKI.

**Results:**

In 257 patients with septic shock, 215 (84%) developed SA-AKI within 48 h of ICU admission, and 111 (43%) progressed to persistent SA-AKI. Patients with persistent SA-AKI had a significantly higher risk of 90-day mortality (adjusted HR, 2.75; 95% CI, 1.39–5.42; *P* = 0.004) and hospital mortality (adjusted OR, 4.29; 95% CI, 1.87–10.70; *P* < 0.001) than those with transient SA-AKI. Greater time-weighted average vasoactive-inotropic score was independently associated with the development of persistent SA-AKI (adjusted OR, 1.03; 95% CI, 1.01–1.06; *P* = 0.03).

**Conclusions:**

In patients with septic shock requiring high-dose norepinephrine, persistent SA-AKI is associated with worse clinical outcomes. These findings support the need for further research on risk stratification and targeted interventions based on the trajectory of SA-AKI.

**Trial registration:**

The study was registered on UMIN Clinical Trial Registry (UMIN000038302) on November 1, 2019.

## Background

Sepsis-associated acute kidney injury (SA-AKI) is a common complication of sepsis [1] and has been consistently associated with poor outcomes, including increased mortality and prolonged ICU stay [2,3]. Beyond its acute impact, the long-term consequences are equally concerning; survivors of SA-AKI are at elevated risk of the development of chronic kidney disease (CKD) or end-stage kidney disease (ESKD) [4]. Despite this substantial disease burden, definitive therapeutic interventions specifically targeting SA-AKI remain elusive. Multiple randomized controlled trials investigating novel pharmacological agents have not demonstrated significant improvements in clinical outcomes [5,6], highlighting the need for patient stratification approaches [7].

Emerging evidence suggests that the dynamic trajectory of SA-AKI may provide critical insights into prognosis. Specifically, persistent or non-resolving AKI patterns are associated with increased mortality and poor renal recovery in patients with sepsis and critical illness [[8], [9], [10], [11], [12]]. However, the association of persistent SA-AKI with clinical outcomes in septic shock has not been well-studied. Septic shock differs from sepsis in its hemodynamic profile and management, including fluid resuscitation and vasopressor use that may affect the development and persistence of SA-AKI [13]. In addition, the unique pathophysiological mechanisms of septic shock—including severe macrocirculatory dysfunction, microvascular shunting, and dysregulated host inflammatory responses—likely influence the progression of AKI [14,15].

To address this knowledge gap, we aimed to investigate the epidemiology of persistent SA-AKI and its association with mortality in septic shock. In addition, we sought to identify hemodynamic risk factors and management-related variables associated with the development of persistent SA-AKI in this population. Understanding these associations could improve risk stratification and inform future clinical trials targeting SA-AKI in septic shock.

## Methods

### Data source and participants

We analyzed data from a multicenter prospective cohort registry—BEst Available Treatment for septic SHOCK (BEAT-SHOCK) registry. Details of the study design, setting, participants, and data collection were described previously [16]. Briefly, the BEAT-SHOCK registry was designed to evaluate the effectiveness of various management strategies for septic shock. A total of 309 adult patients (≥18 years old) with septic shock requiring high-dose norepinephrine (≥0.2 μg/kg/min) within 24 h of sepsis onset were enrolled from the ICUs at 20 hospitals across Japan between January 2020 and December 2022. The threshold of norepinephrine ≥0.2 μg/kg/min was used to focus on patients with severe cardiovascular failure, which aligns with recent literature [17]. Septic shock was defined according to the Sepsis-3 criteria [18]. The registry excluded patients who died or were discharged from the ICU within 48 h of admission, as well as those with pre-existing organ dysfunction before sepsis onset, severe liver cirrhosis (Child-Pugh class C), severe chronic heart failure (New York Heart Association class IV), acute myocardial infarction, or terminal cancer with limited life expectancy. The study was approved by the Tohoku University Hospital Ethics Boards (approval number 2019-1-402) with a waiver of informed consent and registered in the UMIN Clinical Trial Registry on November 1, 2019 (UMIN000038302).

### Definitions and study population

This post-hoc analysis investigated the incidence and clinical outcomes of SA-AKI and persistent SA-AKI. SA-AKI was defined as the presence of both sepsis [18] and AKI as defined by the Kidney Disease: Improving Global Outcomes (KDIGO) criteria [19], as endorsed by the 28th Acute Disease and Quality Initiative [20]. In this study, we focused on early SA-AKI, defined as AKI developing within 48 h of ICU admission. AKI diagnosis was based on KDIGO criteria using both serum creatinine and urine output data, with patients classified as having AKI if they met any of the criteria. CKD was defined as a documented history of CKD recorded at ICU admission. For patients without CKD, baseline serum creatinine was estimated using the Modification of Diet in Renal Disease (MDRD) study equation [21], assuming an estimated glomerular filtration rate (eGFR) of 75 ml/min/1.73 m^2^, as pre-admission creatinine values were unavailable. For patients with CKD, the serum creatinine level at ICU admission was used as the baseline. Patients were classified into persistent SA-AKI, transient SA-AKI, or no SA-AKI. The time of SA-AKI diagnosis (the first time any KDIGO criterion was met after ICU admission) was defined as time zero. Persistent SA-AKI was defined as an episode of AKI by KDIGO criteria (by serum creatinine level, urine output, or ongoing KRT requirement) lasting for at least 48 h after time zero [11]. Transient SA-AKI was defined as SA-AKI that no longer met the KDIGO criteria (by serum creatinine level, urine output, or ongoing KRT requirement) at 48 h after the diagnosis of SA-AKI. Serum creatinine was recorded at ICU admission and at least daily during the first 7 days after ICU admission or until ICU discharge, whichever came first. We excluded patients with end-stage kidney disease or those receiving continuous kidney replacement therapy (CKRT) for non-renal indications.

### Outcomes of interests

The primary outcome of this analysis was 90-day mortality. Secondary outcomes included in-hospital mortality and composite outcome at day 28, defined as 28-day mortality or ongoing KRT dependence on day 28.

### Statistical analyses

Categorical variables were summarized as numbers (percentages), and continuous variables as medians (interquartile ranges). To compare the demographic and clinical characteristics, we used the Kruskal–Wallis test and chi-square test for comparisons involving more than two groups, as appropriate. We used the Kaplan–Meier curves to estimate 90-day mortality across three groups—persistent SA-AKI, transient SA-AKI, and no AKI, and compared them with log-rank test. To compare the association of persistent SA-AKI versus transient SA-AKI on clinical outcomes, we constructed multivariable Cox proportional hazards models (for 90-day mortality) and logistic regression models (for in-hospital mortality and composite outcome at day 28). In the multivariable models, we used predefined confounding factors based on previous literature and clinical relevance, which were age, male sex, body mass index (BMI), Charlson comorbidity index (CCI), and acute physiology and chronic health evaluation (APACHE) II score [12]. To identify risk factors related to hemodynamic status and management associated with the development of persistent SA-AKI, we evaluated the association of time-weighted average (TWA) mean arterial pressure (MAP) and vasoactive-inotropic score (VIS) during the first 48 h, cumulative fluid balance during the first 3 days, and maximum serum lactate levels on day 1 with the development of persistent SA-AKI among patients who developed SA-AKI (persistent SA-AKI vs. transient SA-AKI). TWA MAP and VIS were calculated using 2-h interval data from ICU admission to 48 h, with each measurement weighted by the duration between successive time points. VIS was calculated by the following equation [22]: VIS = norepinephrine (μg/kg/min) × 100 + epinephrine (μg/kg/min) × 100 + dopamine (μg/kg/min) + dobutamine (μg/kg/min) + vasopressin (unit/kg/min) × 10,000 + levosimendan (μg/kg/min) × 50 + milrinone (μg/kg/min) × 10. We constructed multivariable logistic regression models adjusting for age, sex, BMI, CCI, APACHE II score, and use of MV within the first 6 h of ICU admission [12,23].

We performed sensitivity analyses to validate the robustness of the results. First, we investigated the association of persistent severe AKI—defined as a composite of [1] persistent KDIGO stage 3 AKI ≥ 72 h [2], dialysis, or [3] death following KDIGO stage 3 AKI [24]—with mortality outcomes (90-day mortality and in-hospital mortality). Second, we investigated another definition of persistent SA-AKI, in which persistent SA-AKI was defined as an episode of AKI by KDIGO criteria (by serum creatinine level, urine output, or ongoing KRT requirement) lasting for at least 72 h. Third, we excluded patients with CKD to reduce the risk of misclassification due to unavailable baseline serum creatinine levels. Fourth, we used serum creatinine at ICU admission as baseline when it was lower than the estimated baseline creatinine using the MDRD equation. Fifth, we restricted the analysis to patients receiving noradrenaline of >0.4 μg/kg/min based on the literature [17]. Sixth, we added statistically significant characteristics to the multivariable model to identify risk factors related to hemodynamic status and management associated with the development of persistent SA-AKI.

We conducted the statistical analysis by using R version 4.3.2 (R Foundation, Vienna, Austria). All P values were two-tailed, with *P* < 0.05 considered statistically significant.

## Results

### Incidence and characteristics of persistent SA-AKI

Of the 309 patients included in the BEAT-SHOCK registry, the current post-hoc analysis included 257 patients with septic shock (Figure S1). The median age was 72 years old (interquartile range [IQR] 65−81 years old), and 55% were male (Table 1). The median SOFA and APACHE II scores were 11 and 26, respectively. SA-AKI developed within the first 48 hours of ICU admission in 84% of patients (215/257), of whom 52% subsequently developed persistent SA-AKI. In contrast, only 3 patients (1%) developed late SA-AKI (onset between 48 h and 7 days), and therefore our analyses focused on early SA-AKI. While 76% of patients with persistent SA-AKI were classified as KDIGO stage 3 within 48 h, only 24% of those with transient SA-AKI were classified as KDIGO stage 3 within 48 h. There were significant differences in BMI, infection source, SOFA score, APACHE II score, and serum creatinine level among the three groups—persistent SA-AKI, transient SA-AKI, and no SA-AKI (all *P* < 0.05). Regarding the source of infection, urinary tract infection was significantly more frequent in patients with persistent SA-AKI than in those with no SA-AKI (23% vs. 5%, *P* = 0.02) in post-hoc pairwise comparisons with Bonferroni correction. Among patients with persistent SA-AKI, 55 patients (50%) received RRT at 48 h after the diagnosis of SA-AKI.

**Table 1:** Baseline characteristics of patients with septic shock stratified by the pattern of SA-AKI.

	Total (N = 257)	Persistent SA-AKI (N = 111)	Transient SA-AKI (N = 104)	No SA-AKI (N = 42)	P value
Age (years), median (IQR)	72 (65–81)	73 (67–82)	73 (65–80)	71 (62–78)	0.55
Male sex	141 (55)	64 (58)	56 (54)	21 (50)	0.67
BMI (kg/m^2^), median (IQR)	22 (20–26)	24 (20–27)	22 (20–25)	21 (19–24)	0.01
Coexisting conditions					
Chronic heart disease	17 (7)	11 (10)	4 (4)	2 (5)	0.18
Chronic respiratory disease	4 (2)	2 (2)	2 (2)	0 (0)	0.67
Liver failure	3 (1)	0 (0)	2 (2)	1 (2)	0.31
Chronic kidney disease	9 (4)	4 (4)	3 (3)	2 (5)	0.85
Immunodeficiency	13 (5)	6 (5)	4 (4)	3 (7)	0.70
Diabetes mellitus	38 (15)	22 (20)	11 (11)	5 (12)	0.14
Charlson Comorbidity Index, median (IQR)	1 (0−2)	1 (0–3)	1 (0–2)	1 (0–2)	0.40
**Admission source**					0.23
Emergency department	186 (72)	75 (68)	81 (78)	30 (71)	
General ward	25 (10)	10 (9)	8 (8)	7 (17)	
Other intensive care unit	5 (2)	4 (4)	1 (1)	0 (0)	
Other hospital	41 (16)	22 (20)	14 (14)	5 (12)	
Infection					
Blood culture positivity	131 (51)	69 (62)	50 (48)	12 (29)	0.001
Source of infection					0.03
Abdomen	95 (37)	35 (32)	40 (39)	20 (48)	
Urinary tract	44 (17)	26 (23)	16 (15)	2 (5)	
Chest	52 (20)	21 (19)	18 (17)	13 (31)	
Skin and tissue	43 (17)	22 (20)	16 (15)	5 (12)	
Other	23 (9)	7 (6)	14 (14)	2 (5)	
Severity					
SOFA score, median (IQR)	11 (9–13)	13 (11–14)	10 (9–12)	10 (8–11)	<0.001
APACHE II score, median (IQR)	26 (21–32)	29 (25–35)	24 (20–29)	25 (17–29)	<0.001
Vital signs and laboratory data on ICU admission					
MAP (mmHg), median (IQR)	69 (57–81)	70 (57–78)	66 (56–80)	73 (64–87)	0.05
HR (/min), median (IQR)	110 (93–121)	110 (90–120)	110 (94–125)	111 (97–117)	0.85
Lactate (mmol/L), median (IQR)	4.0 (2.5–6.5)	4.5 (2.6–7.7)	4.0 (2.6–5.6)	3.2 (2.3–5.4)	0.10
Creatinine (mg/dL), median (IQR)	1.8 (1.1–2.8)	2.6 (1.8–3.5)	1.6 (1.1–2.2)	0.9 (0.7–1.1)	<0.001
Organ support					
Mechanical ventilation within first 6 h after ICU admission	190 (74)	84 (76)	70 (67)	36 (86)	0.06
Max noradrenaline within first 6 h after ICU admission (μg/kg/min), median (IQR)	0.30 (0.20–0.40)	0.30 (0.20–0.40)	0.27 (0.20–0.34)	0.30 (0.25–0.40)	0.14
Vasopressin use within first 6 h after ICU admission	164 (64)	75 (68)	60 (58)	29 (69)	0.24
Max vasopressin within first 6 h after ICU admission (μg/kg/min), median (IQR)	1.50 (0.00–2.00)	1.50 (0.00–2.00)	1.00 (0.00–2.00)	1.25 (0.00–2.00)	0.47
Max VIS within first 6 h after ICU admission, median (IQR)	34 (24–45)	35 (25–46)	30 (24–42)	34 (28–47)	0.14
KRT within 7 days	75 (29)	66 (60)	9 (9)	0 (0)	<0.001
AKI KDIGO stage					<0.001
1	39 (15)	7 (6)	32 (31)	0 (0)	
2	67 (26)	20 (18)	47 (45)	0 (0)	
3	109 (42)	84 (76)	25 (24)	0 (0)	
Intravenous contrast administration	121 (47)	44 (40)	52 (50)	25 (60)	0.07
Surgical intervention	90 (35)	34 (31)	35 (34)	21 (50)	0.08

Data are presented as median [interquartile range] or N (%). Variables were compared using Kruskal-Wallis test or chi-square test as appropriate.

Abbreviations: AKI, acute kidney injury; APACHE, Acute Physiology and Chronic Health Evaluation; BMI, body mass index; HR, heart rate; ICU, intensive care unit; IQR, interquartile range; KDIGO, Kidney Disease: Improving Global Outcomes; KRT, kidney replacement therapy; MAP, mean arterial pressure; SA-AKI, sepsis-associated acute kidney injury; SOFA, Sequential Organ Failure Assessment; VIS, vasoactive-inotropic score.

### Association of persistent SA-AKI with clinical outcomes

**Fig. 1:**
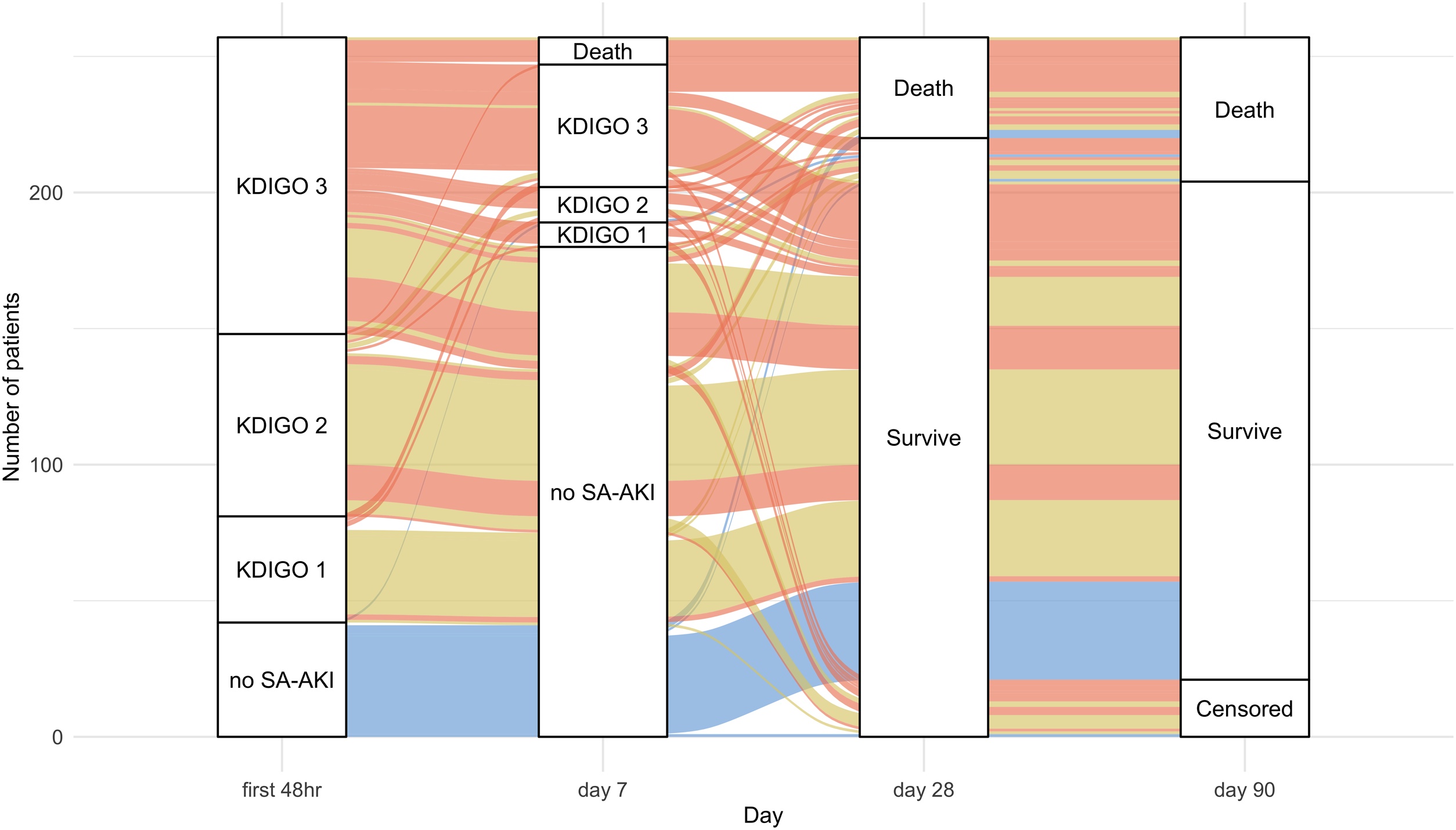
Trajectory of kidney injury and mortality. The x-axis indicates the timepoints from ICU admission (first 48 h, day 7, day 28, and day 90), and the y-axis shows the number of patients. Each colored ribbon represents patient trajectories based on SA-AKI persistence: red indicates persistent SA-AKI, yellow indicates transient SA-AKI, and blue indicates no SA-AKI. Patients censored at 90 days (n = 21, 8%) were lost to follow-up. Most patients with persistent SA-AKI were classified as KDIGO stage 3 within the first 48 h and continued to have SA-AKI at day 7. In contrast, most patients with transient SA-AKI had no SA-AKI at day 7. Abbreviations: KDIGO, Kidney Disease Improving Global Outcomes; SA-AKI, sepsis-associated acute kidney injury.

**Fig. 2:**
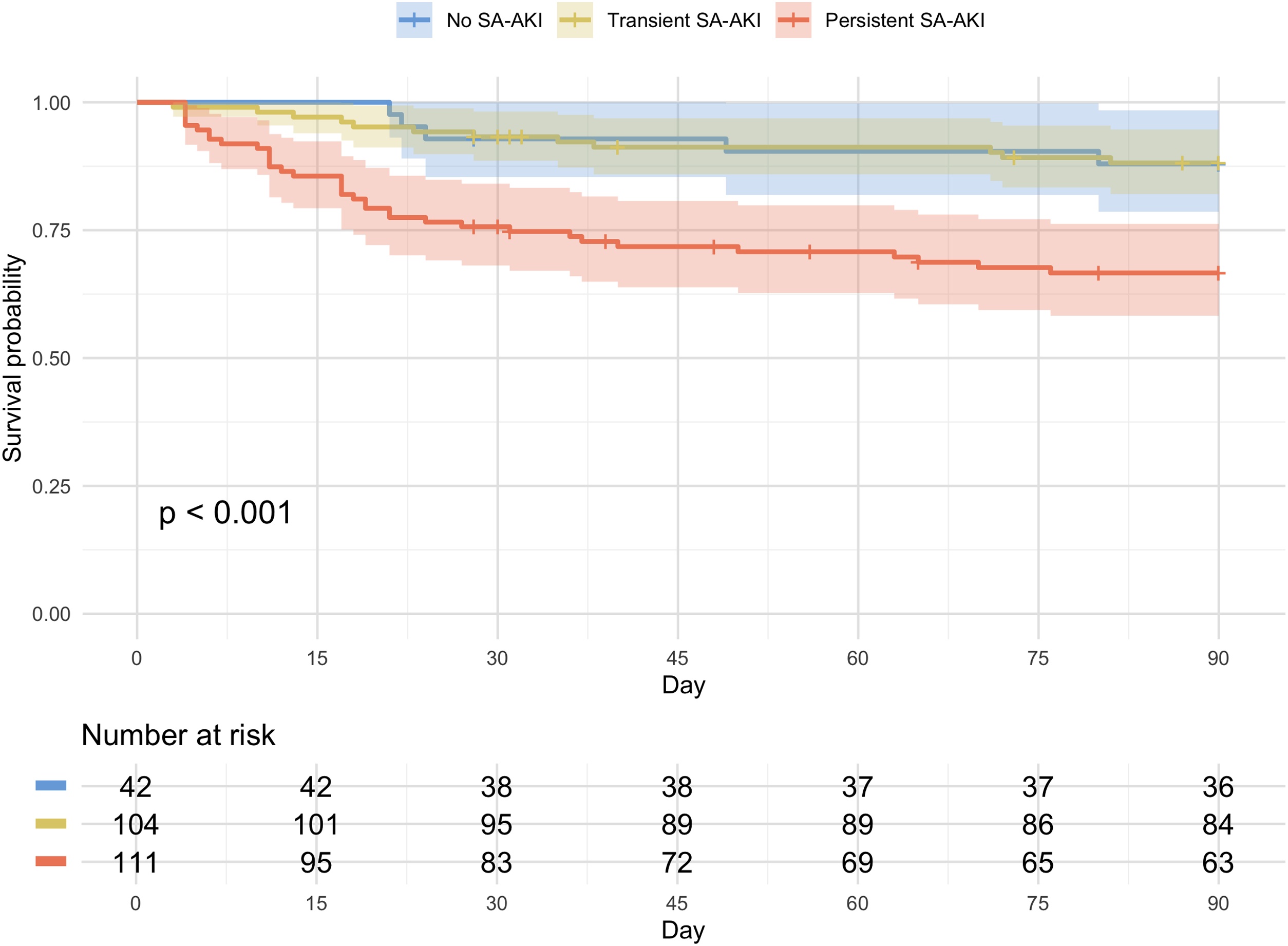
Kaplan–Meier curves for 90-day mortality, according to the trajectory of SA-AKI. Kaplan-Meier curves for 90-day mortality were significantly different across the three SA-AKI trajectories (P < 0.001). Abbreviation: SA-AKI, sepsis-associated with acute kidney injury.

Overall, 19% of patients died in hospital, and the 90-day cumulative mortality rate was 21% (Table 2). Among patients with persistent SA-AKI, most continued to have AKI or had died by day 7, and the 90-day cumulative mortality rate reached 33% (Fig. 1 and Table 2). In contrast, most patients with transient SA-AKI no longer met AKI criteria at day 7, with a 90-day cumulative mortality rate of only 12%. In post hoc pairwise comparisons with Bonferroni correction, 90-day mortality in patients with transient SA-AKI was not significantly different from that in patients with no SA-AKI (12% vs. 12%, P = 1.00). The 90-day cumulative mortality rates were significantly different across the three SA-AKI trajectories—persistent SA-AKI, transient SA-AKI, and no SA-AKI, with the highest mortality in the persistent SA-AKI group (*P* < 0.001; Fig. 2). In sensitivity analysis using serum creatinine at ICU admission as baseline when it was lower than MDRD-estimated values (Table S5), results were consistent. In multivariable Cox proportional hazard models, patients with persistent SA-AKI had a significantly higher risk of 90-day mortality than those with transient SA-AKI (adjusted hazard ratio [aHR], 2.75; 95% confidence interval [CI], 1.39–5.42; *P* = 0.004; Table 3). Likewise, persistent SA-AKI was associated with increased risks of in-hospital mortality (adjusted odds ratio [aOR], 4.29; 95% CI, 1.87–10.70; *P* < 0.001) and composite outcome at day 28—defined as 28-day mortality or ongoing KRT dependence on day 28 (28% vs. 7%; aOR, 4.10; 95% CI, 1.70–11.05; *P* = 0.003). In a sensitivity analysis comparing patients with persistent severe versus transient severe SA-AKI, baseline characteristics and clinical outcomes according to this alternative definition are summarized in Tables S1 and S2. Patients with persistent severe SA-AKI had significantly higher risks of 90-day mortality (39% vs. 21%; aHR, 2.24; 95% CI, 1.17–4.29; *P* = 0.02; Table S3) and in-hospital mortality (39% vs. 18%; aOR, 3.20; 95% CI, 1.36–7.66; *P* = 0.01). Additional sensitivity analyses using a 72-hour trajectory to define persistent SA-AKI, excluding patients with CKD, applying an alternative definition of baseline criteria, and restricting the cohort to patients receiving noradrenaline of >0.4 μg/kg/min showed consistent results with the main analyses (Tables S4–S7).

**Table 2:** Outcomes of patients with septic shock stratified by the pattern of SA-AKI.

	Total (N = 257)	Persistent SA-AKI (N = 111)	Transient SA-AKI (N = 104)	No SA-AKI (N = 42)	P value
ICU mortality	26 (10)	20 (18)	4 (4)	2 (5)	0.001
In-hospital mortality	49 (19)	35 (32)	9 (9)	5 (12)	<0.001
28-day mortality	37 (14)	27 (24)	7 (7)	3 (7)	<0.001
90-day cumulative mortality†	53 (21)	36 (32)	12 (12)	5 (12)	<0.001
AKI KDIGO stage at day 7‡					<0.001
1	9 (5)	9 (11)	0 (0)	0 (0)	
2	13 (8)	10 (13)	2 (3)	1 (5)	
3	38 (23)	36 (45)	2 (3)	0 (0)	
Use for KRT at day 28	4 (2)	4 (4)	0 (0)	0 (0)	0.07
Length-of-ICU stay, median (IQR)	8 (5–14)	9 (6–14)	8 (5–13)	6 (5–12)	0.12
Length-of-hospital stay, median (IQR)	28 (18–60)	26 (17–60)	30 (19–63)	31 (22–57)	0.53

Data are presented as median [interquartile range] or N (%). Variables were compared using Kruskal-Wallis test or chi-square test as appropriate.

Abbreviations: ICU, intensive care unit; IQR, interquartile range; KRT, kidney replacement therapy; SA-AKI, sepsis-associated acute kidney injury.

† Kaplan-Meier estimate showing the number of deaths (cumulative mortality). A total of 21 patients (8%) were lost to follow-up at 90 days.

‡ AKI KDIGO stage at day 7 was evaluated only among patients who were alive and stayed in ICU at day 7. 80 patients in persistent AKI, 65 patients in transient SA-AKI, and 20 patients in no SA-AKI were evaluated.

**Table 3:** Association of persistent SA-AKI with outcomes.

Primary outcome	Crude hazard ratio (95% CI)	*P* value	Adjusted hazard ratio (95% CI)	*P* value
90-day mortality	3.38 (1.76–6.50)	<0.001	2.75 (1.39–5.42)	0.004

Secondary outcomes	Crude odds ratio (95% CI)	*P* value	Adjusted odds ratio (95% CI)	*P* value

In-hospital mortality	4.86 (2.29–11.34)	<0.001	4.29 (1.87–10.70)	<0.001
Composite outcome at day 28†	5.37 (2.37–13.86)	<0.001	4.10 (1.70–11.05)	0.003

Cox proportional hazard model for 90-day mortality was fit, adjusted for age, sex, BMI, APACHE II score, and Charlson comorbidity index. Logistic regression models for in-hospital mortality and composite outcome at day 28 were fit, adjusted for age, sex, BMI, APACHE II score, and Charlson comorbidity index.

All comparisons were made between patients with persistent SA-AKI and those with transient SA-AKI, which served as the reference group.

Abbreviations: APACHE acute physiology and chronic health evaluation; BMI, body mass index; CI, confidence interval; KRT, kidney replacement therapy; SA-AKI, sepsis-associated acute kidney injury.

† Defined as 28-day mortality or ongoing KRT dependence on day 28.

### Risk factors for persistent SA-AKI

**Fig. 3:**
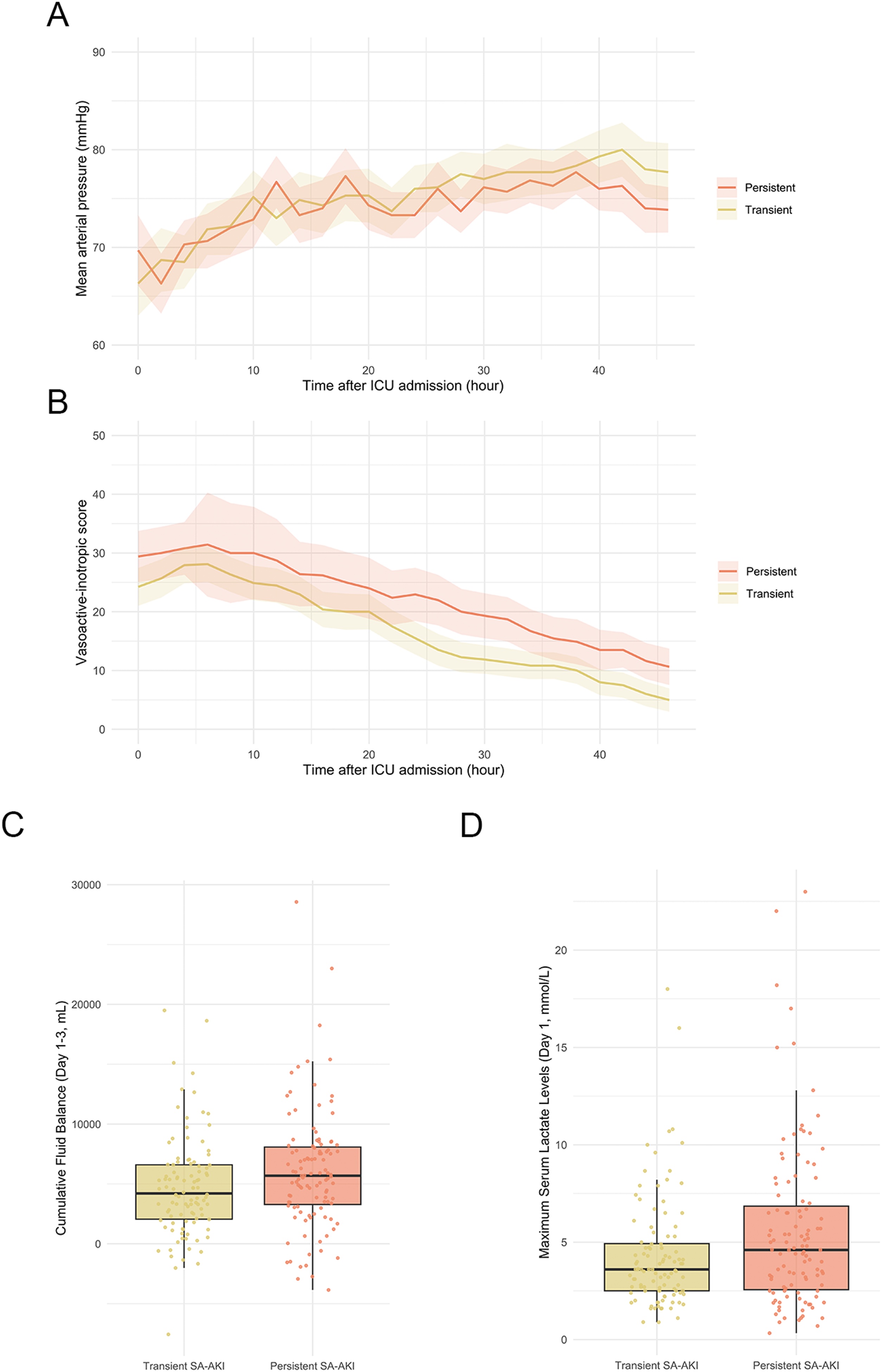
Association of factors related to hemodynamic status and management with the development of persistent SA-AKI. **(A)** Mean arterial pressure (MAP) over the first 48 h after ICU admission. TWA MAP did not significantly differ between groups in a multivariable logistic regression model (adjusted OR 1.00, 95% CI 0.96–1.04, p = 0.95; Table 4). **(B)** Vasoactive-Inotropic Score (VIS) over the first 48 h. TWA VIS significantly differed between groups in a multivariable logistic regression model (adjusted OR 1.03, 95% CI 1.01–1.06, p = 0.03; Table 4). **(C)** Cumulative fluid balance from day 1 to day 3. Cumulative fluid balance did not significantly differ between groups in a multivariable logistic regression model (adjusted OR 1.01 per 100 mL, 95% CI 1.00–1.01, p = 0.13; Table 4). **(D)** Maximum serum lactate level on day 1. Serum lactate level did not significantly differ between groups in a multivariable logistic regression model (adjusted OR 1.03, 95% CI 0.95–1.14, p = 0.48; Table 4). Abbreviations: CI, confidence interval; MAP, mean arterial pressure; OR, odds ratio; SA-AKI, sepsis-associated with acute kidney injury; TWA, time-weighted average; VIS, Vasoactive-Inotropic Score.

We investigated the associations of several factors related to hemodynamic status and management—including MAP, VIS, cumulative fluid balance, and serum lactate level— with the development of persistent SA-AKI. In univariable logistic regression models, multiple factors including higher VIS, greater cumulative fluid balance, and elevated serum lactate level were significantly associated with the development of persistent SA-AKI (All *P* < 0.05; Table 4 and Fig. 3). In multivariable logistic regression models, higher VIS remained significantly associated with the development of persistent SA-AKI (aOR, 1.03; 95% CI, 1.01–1.06; *P* = 0.03; Table 4). In addition, higher APACHE II score (aOR, 1.09; 95% CI, 1.04–1.14; *P* < 0.001) and greater BMI (aOR, 1.09; 95% CI, 1.02–1.17; *P* = 0.01) were also significantly associated with the development of persistent SA-AKI. In a sensitivity analysis including blood culture positivity and source of infection, these associations were consistent (Table S6).

**Table 4:** Adjusted associations of factors related to hemodynamic status and management with the development of persistent SA-AKI.

Variable	Univariable OR (95% CI)	*P* value	Multivariable OR (95% CI)	*P* value
TWA MAP during the first 48 h	0.98 (0.95–1.01)	0.21	1.00 (0.96–1.04)	0.95
TWA VIS during the first 48 h	1.04 (1.02–1.06)	0.001	1.03 (1.01–1.06)	0.03
Cumulative fluid balance by day 3, per 100 ml	1.01 (1.00–1.01)	0.04	1.01 (1.00–1.01)	0.13
Maximum serum lactate level on day 1	1.10 (1.02–1.20)	0.02	1.03 (0.95–1.14)	0.48
Age	1.00 (0.98–1.03)	0.71	1.01 (0.98–1.03)	0.54
Male sex	1.17 (0.68–2.00)	0.57	0.85 (0.45–1.58)	0.61
BMI	1.08 (1.02–1.14)	0.01	1.09 (1.02–1.17)	0.01
CCI	1.12 (0.94–1.34)	0.20	1.02 (0.84–1.25)	0.81
APACHE II score	1.10 (1.06–1.15)	<0.001	1.09 (1.04–1.14)	<0.001
MV within the first 6 h of ICU admission	1.51 (0.83–2.76)	0.17	0.99 (0.49–1.99)	0.97

A multivariable logistic regression model was fitted to evaluate the association of hemodynamic status and management-related factors—including MAP, VIS, cumulative fluid balance, and serum lactate level—with the development of persistent SA-AKI. TWA MAP and VIS were calculated using 2 -h interval data from ICU admission to 48 h, with each measurement weighted by the duration between successive time points. The model was adjusted for age, sex, BMI, CCI, APACHE II score, and use of MV within the first 6 h of ICU admission.

Abbreviations: APACHE, acute physiology and chronic health evaluation; BMI, body mass index; CCI, Charlson Comorbidity Index; ICU, intensive care unit; MAP, mean arterial pressure; MV, mechanical ventilation; SA-AKI, sepsis-associated acute kidney injury; TWA, time-weighted average; VIS, vasoactive-inotropic score.

## Discussion

Based on the analysis of a prospective multicenter registry of patients with septic shock who received high-dose norepinephrine, we found that 84% had SA-AKI within 48 h of ICU admission, and 43% of all patients developed persistent SA-AKI (accounting for 52% of those with SA-AKI). Persistent SA-AKI was significantly associated with increased 90-day mortality. In addition, a higher VIS was independently associated with the development of persistent SA-AKI. Our findings suggest that persistent SA-AKI is a common and clinically significant phenotype in septic shock that is independently associated with mortality.

Emerging evidence, consistent with our findings, have identified significant associations of SA-AKI trajectory with mortality. For example, a single-center prospective study in a surgical ICU focusing on sepsis reported that patients with persistent SA-AKI without renal recovery had a higher risk of all-cause mortality than those without SA-AKI [11]. Similarly, a prospective multicenter cohort study in patients with sepsis using RIFLE criteria reported that patients with persistent SA-AKI were associated with an increased risk of mortality by day 30 and up to 1 year than those with no SA-AKI [9]. Although the reported incidence of persistent SA-AKI varies widely—from 12% to 68% [11,12]—likely due to variability in definitions and populations studied, the consistent relationship between persistent SA-AKI and mortality underscores the clinical relevance of persistent SA-AKI in patients with septic shock. Importantly, the independent association between persistent SA-AKI and mortality after adjusting for disease severity suggests that AKI duration itself represents a clinically meaningful trajectory. However, whether persistent SA-AKI represents a therapeutic target remains to be determined in further studies.

Resuscitative therapy and hemodynamic monitoring—including maintenance of blood pressure, catecholamine use, fluid management, and serum lactate levels— are central to the clinical management of septic shock, as highlighted in recent guidelines [13]. However, the relationship between these factors and the development of persistent AKI remains poorly understood. Notably, although both groups maintained similar MAP values—typically above 65 mmHg—during the first 48 h, vasopressor dependency, as quantified by VIS, was independently associated with the development of persistent SA-AKI. Consistent with our findings, a prior study reported that maximum VIS was associated with the development of severe AKI in children with sepsis [25]. While norepinephrine effectively increases systemic blood pressure and renal blood flow, experimental studies suggest it may exacerbate renal medullary hypoxia through increased oxygen consumption [26,27]. These findings align with recent randomized controlled trials examining the optimal hemodynamic targets in septic shock. One trial showed no significant association between higher MAP targets (80−85 mmHg vs. 65−70 mmHg) and reduced need for kidney replacement therapy after AKI [28]. More recently, another randomized controlled trial demonstrated that permissive hypotension (MAP 60−65 mmHg) was safe and potentially beneficial, importantly allowing for reduced vasopressor exposure [29]. Collectively, these findings suggest that current hemodynamic management strategies—focused primarily on MAP targets—may be insufficient to prevent persistent SA-AKI, likely due to the complex relationship between systemic hemodynamics and renal microcirculatory function. In our multivariable analysis, cumulative fluid balance was not significantly associated with the development of persistent SA-AKI. While previous studies have demonstrated that cumulative fluid balance was associated with impaired renal recovery in patients with AKI [12,30], results from recent trials have remained conflicting: the CLASSIC trial reported no clinical benefit of fluid restriction for patients with septic shock [31]. Therefore, the optimal fluid management strategy to prevent persistent SA-AKI warrants further investigation. The current study builds on these prior reports and extends them by focusing on patients with septic shock at particularly high risk for persistent kidney dysfunction. However, whether management of VIS can prevent the development of persistent SA-AKI warrants further investigation.

This study has several limitations. First, the sample size was relatively small. The consistency of the findings across sensitivity analyses supported the findings with statistical significance. Second, renal outcomes at day 90 were not assessed due to the lack of long-term follow-up data. Third, the absence of baseline serum creatinine, particularly in patients with underlying chronic kidney disease, may have led to misclassification of AKI status. Specifically, in CKD patients, the use of ICU admission creatinine as baseline does not capture AKI present on admission. Additionally, in this study, CKD was identified based on a documented comorbidity at ICU admission according to the registry manual, and pre-admission creatinine values to verify chronicity were not available; therefore, CKD may have been under-ascertained. However, the proportion of CKD patients was small (4%), and a sensitivity analysis excluding these patients showed consistent results. Fourth, as with any observational study, our causal inference could be confounded by unmeasured factors. Finally, immortal time bias is a potential concern because patients who died within 48 h of ICU admission were excluded. More importantly, this exclusion may have introduced selection bias by systematically excluding patients who died early in their ICU course.

## Conclusions

The analysis of a prospective multicenter registry of patients with septic shock demonstrates a higher incidence of persistent SA-AKI in septic shock and its significant association of persistent SA-AKI with mortality. In addition, VIS was identified as a key risk factor for the development of persistent SA-AKI. Our observations suggest that early recognition of persistent SA-AKI may facilitate rapid identification of patients at high risk of mortality. Future studies focusing on persistent SA-AKI are warranted to clarify whether persistent SA-AKI represents a modifiable target for therapeutic intervention and could improve clinical outcomes.

## CRediT authorship contribution statement

MK carried out the statistical analysis and drafted the initial manuscript. YK, TM, HY, and KM designed the study. TM served as the study statistician and was responsible for the methodology, data curation, and statistical analysis. YK, TM, HY, KM, NM, YO, KK, SO, NH, and NS revised the initial manuscript. All authors read and approved the final manuscript.

## Consent for publication

The ethics committee of Tohoku University Hospital (approval number 2019-1-402) and the ethics committees of all other participating hospitals approved this study with an opt-out policy.

## Ethics approval and consent to participate

The ethics committee of Tohoku University Hospital (approval number 2019-1-402) and the ethics committees of all other participating hospitals approved this study with a waiver of informed consent and an opt-out policy in accordance with the Ethical Guidelines for Medical and Biological Research Involving Human Subjects.

## Declaration of Generative AI and AI-assisted technologies in the writing process

During the preparation of this work the authors used Claude in order to generate R code and perform grammar checking for the manuscript. After using this tool/service, the authors reviewed and edited the content as needed and take full responsibility for the content of the published article.

## Funding

The Institute for Clinical Effectiveness (ICE) served as the independent data center that managed the data collection, monitoring, validation, and statistical analyses of the BEAT-SHOCK registry. ICE received a contracted grant from Toray Industries Inc., Tokyo, Japan. This post-hoc analysis was supported by JSPS KAKENHI (Grant Number 25K12297).

## Availability of data and materials

The datasets used and/or analyzed during the current study are available from the corresponding author on reasonable request.

## Declaration of competing interest

Dr. Kyo reports lecturer's fee from TXP Medical. Dr. Kawazoe reports lecturer’s fees from Toray Industries Inc. and Asahi Kasei Pharma. Dr. Morimoto reports lecturer's fees from Abbott, Boehringer Ingelheim, Bristol-Myers Squibb, Daiichi Sankyo, Eisai, Pfizer and UCB; advisory board for GlaxoSmithKline. Dr. Miyamoto reports lecturer's fees from Asahi Kasei Pharma, Japan Blood Products Organization, Chugai Pharmaceutical, and Toray Industries Inc.
