# Corrigendum to “Clinical outcomes of persistent sepsis-associated acute kidney injury in septic shock: a post-hoc analysis of a multicenter prospective cohort study” [Ann Intensive Care (2026) 100041]

**DOI:** 10.1016/j.aicoj.2026.100065

**Published:** 2026-04-24

**Authors:** Michihito Kyo, Yu Kawazoe, Takeshi Morimoto, Hitoshi Yamamura, Kyohei Miyamoto, Noriko Miyagawa, Yoshinori Ohta, Kazuya Kikutani, Shinichiro Ohshimo, Nobuyuki Hirohashi, Nobuaki Shime

**Affiliations:** a Department of Emergency and Critical Care Medicine, Graduate School of Biomedical and Health Sciences, Hiroshima University, Hiroshima, Japan; b Department of Radiation Disaster Medicine, Research Institute for Radiation Biology and Medicine, Hiroshima University, Hiroshima, Japan; c Department of Emergency and Critical Care Medicine, Sendai Medical Center, Sendai, Japan; d Department of Data Science, Hyogo Medical University, Nishinomiya, Japan; e Department of Emergency and Critical Care Medicine, Japan Community Health Care Organization, Osaka Minato Central Hospital, Osaka, Japan; f Department of Emergency and Critical Care Medicine, Wakayama Medical University, Wakayama, Japan; g Department of Emergency and Critical Care Medicine, National Hospital Organization Kyoto Medical Center, Kyoto, Japan

The authors regret that affiliations a and b were switched and apologize for the inconvenience caused.

