# Supplementary material for: Clinical outcomes of persistent sepsis-associated acute kidney injury in septic shock: a post-hoc analysis of a multicenter prospective cohort study
Source: Ann Intensive Care. 2026 Feb 27;16:100041. doi: 10.1016/j.aicoj.2026.100041 (PMC13045545; doi:10.1016/j.aicoj.2026.100041)
Supplement: Supplementary file 1 [file mmc1.docx]

**Supplementary Materials**

**Clinical outcomes of persistent sepsis-associated acute kidney injury in septic shock: a post-hoc analysis of a multicenter prospective cohort study**

**Authors:** Michihito Kyo, MD, PhD; Yu Kawazoe, MD, PhD; Takeshi Morimoto, MD, MPH, PhD; Hitoshi Yamamura, MD, PhD; Kyohei Miyamoto, MD, PhD; Noriko Miyagawa, MD; Yoshinori Ohta, MD, PhD; Kazuya Kikutani, MD, PhD; Shinichiro Ohshimo, MD, PhD; Nobuyuki Hirohashi, MD, PhD; Nobuaki Shime, MD, PhD

**Table of Contents**

**Table S1.** Baseline characteristics of patients stratified by the patterns of SA-AKI based on an alternative definition in a sensitivity analysis

**Table S2.** Outcomes of patients stratified by the patterns of SA-AKI based on an alternative definition in a sensitivity analysis

**Table S3.** Association of persistent severe SA-AKI defined by an alternative definition with outcomes

**Table S4.** Association of persistent SA-AKI defined using a 72-hour threshold with outcomes

**Table S5.** Association of persistent SA-AKI with outcomes in patients without pre-existing CKD

**Table S6.** Outcomes of patients with septic shock stratified by the pattern of SA-AKI using the different criteria for estimating baseline creatinine level

**Table S7.** Association of persistent SA-AKI with outcomes in patients receiving noradrenaline >0.4 μg/kg/min

**Table S8.** Adjusted associations of factors related to hemodynamic status and management with the development of persistent SA-AKI in different models in sensitivity analysis

**Figure S1.** Patient flow

**Table S1. Baseline characteristics of patients stratified by the patterns of SA-AKI based on an alternative definition in a sensitivity analysis**

|  | **Persistent severe SA-AKI (N=49)** | **Transient severe SA-AKI (N=127)** | **Stage 1 SA-AKI (N=39)** | **No SA-AKI (N=42)** | **P value** |
| --- | --- | --- | --- | --- | --- |
| Age (years), median (IQR) | 71 (60–78) | 75 (68–83) | 72 (62–80) | 71 (62–78) | 0.02 |
| Male sex | 28 (57) | 70 (55) | 22 (56) | 21 (50) | 0.91 |
| BMI (kg/m^2^), median (IQR) | 24 (21–27) | 22 (20–26) | 21 (20–24) | 21 (19–24) | 0.01 |
| **Coexisting conditions** |  |  |  |  |  |
| Chronic heart disease | 3 (6) | 9 (7) | 3 (8) | 2 (5) | 0.95 |
| Chronic respiratory disease | 0 (0) | 3 (2) | 1 (3) | 0 (0) | 0.53 |
| Liver failure | 0 (0) | 1 (1) | 1 (3) | 1 (2) | 0.59 |
| Chronic kidney disease | 2 (4) | 5 (4) | 0 (0) | 2 (5) | 0.63 |
| Immunodeficiency | 2 (4) | 6 (5) | 2 (5) | 3 (7) | 0.92 |
| Diabetes mellitus | 7 (14) | 21 (17) | 5 (13) | 5 (12) | 0.87 |
| Charlson Comorbidity Index, median (IQR) | 1 (0–2) | 1 (0–3) | 1 (0–2) | 1 (0–2) | 0.48 |
| **Admission source** |  |  |  |  | 0.40 |
| Emergency department | 32 (65) | 31 (80) | 93 (73) | 30 (71) |  |
| General ward | 3 (6) | 3 (8) | 12 (9) | 7 (17) |  |
| Other intensive care unit | 2 (4) | 0 (0) | 3 (2) | 0 (0) |  |
| Other hospital | 12 (25) | 5 (13) | 19 (15) | 5 (12) |  |
| **Infection** |  |  |  |  |  |
| Blood culture positivity | 29 (59) | 64 (50) | 26 (67) | 12 (29) | 0.003 |
| Source of infection |  |  |  |  | 0.02 |
| Abdomen | 10 (20) | 48 (38) | 17 (44) | 20 (48) |  |
| Urinary tract | 15 (31) | 24 (19) | 3 (8) | 2 (5) |  |
| Chest | 12 (25) | 22 (17) | 5 (13) | 13 (31) |  |
| Skin and tissue | 8 (16) | 21 (17) | 9 (23) | 5 (12) |  |
| Other | 4 (8) | 12 (9) | 5 (13) | 2 (5) |  |
| **Severity** |  |  |  |  |  |
| SOFA score, median (IQR) | 13 (11–15) | 12 (10–14) | 9 (8–11) | 10 (8–11) | <0.001 |
| APACHE II score, median (IQR) | 30 (27–36) | 27 (22–33) | 22 (18–27) | 25 (17–29) | <0.001 |
| **Vital signs and laboratory data on ICU admission** |  |  |  |  |  |
| MAP (mmHg), median (IQR) | 70 (63–82) | 65 (55–78) | 71 (61–78) | 73 (64–87) | 0.04 |
| HR (/min), median (IQR) | 110 (88–121) | 109 (90–122) | 110 (98–124) | 111 (97–117) | 0.77 |
| Lactate (mmol/L), median (IQR) | 4.1 (2.4–7.4) | 4.3 (3.0–6.5) | 3.9 (2.5–5.4) | 3.2 (2.3–5.4) | 0.24 |
| Creatinine (mg/dL), median (IQR) | 3.1 (2.0–4.1) | 2.1 (1.4–2.9) | 1.3 (1.1–1.7) | 0.9 (0.7–1.1) | <0.001 |
| **Organ support** |  |  |  |  |  |
| Mechanical ventilation within first 6h after ICU admission | 36 (74) | 94 (74) | 24 (62) | 36 (86) | 0.11 |
| Max Noradrenaline within first 6h after ICU admission (µg/kg/min), median (IQR) | 0.30 (0.20–0.44) | 0.30 (0.20–0.38) | 0.25 (0.20–0.30) | 0.30 (0.25–0.40) | 0.06 |
| Vasopressin use within first 6h after ICU admission | 34 (69) | 82 (65) | 19 (49) | 29 (69) | 0.17 |
| Max vasopressin within first 6h after ICU admission (µg/kg/min), median (IQR) | 1.60 (0.00–2.00) | 1.50 (0.00–2.00) | 0.00 (0.00–2.00) | 1.25 (0.00–2.00) | 0.24 |
| Max VIS within first 6h after ICU admission, median (IQR) | 41 (25–53) | 34 (24–46) | 28 (20–34) | 34 (28–47) | 0.04 |
| KRT within 7 days | 46 (94) | 27 (21) | 2 (5) | 0 (0) | <0.001 |
| AKI KDIGO stage |  |  |  |  | <0.001 |
| 1 | 0 (0) | 0 (0) | 39 (100) | 0 (0) |  |
| 2 | 0 (0) | 67 (53) | 0 (0) | 0 (0) |  |
| 3 | 49 (100) | 60 (47) | 0 (0) | 0 (0) |  |
| Intravenous contrast administration | 20 (41) | 53 (42) | 23 (59) | 25 (60) | 0.07 |
| Surgical intervention | 11 (22) | 15 (39) | 43 (34) | 21 (50) | 0.05 |

Data are presented as median [interquartile range] or N (%). Variables were compared using Kruskal-Wallis test or chi-square test as appropriate.

This table presents a sensitivity analysis using an alternative definition of persistent severe SA-AKI—defined as a composite of (1) persistent KDIGO stage 3 AKI≥72 h, (2) dialysis, or (3) death following KDIGO stage 3 AKI. Transient severe SA-AKI was defined as patients with stage 2–3 AKI within 48 hours of ICU admission who did not meet the criteria for persistent severe SA-AKI.

Abbreviations: AKI, acute kidney injury; APACHE, Acute Physiology and Chronic Health Evaluation; BMI, body mass index; HR, heart rate; ICU, intensive care unit; IQR, interquartile range; KDIGO, Kidney Disease: Improving Global Outcomes; KRT, kidney replacement therapy; MAP, mean arterial pressure; SA-AKI, sepsis-associated acute kidney injury; SOFA, Sequential Organ Failure Assessment; VIS, vasoactive-inotropic score.

**Table S2. Outcomes of patients stratified by the patterns of SA-AKI based on an alternative definition in a sensitivity analysis**

|  | **Persistent severe SA-AKI (N=49)** | **Transient severe SA-AKI (N=127)** | **Stage 1 SA-AKI (N=39)** | **No SA-AKI (N=42)** | **P value** |
| --- | --- | --- | --- | --- | --- |
| ICU mortality | 8 (16) | 16 (13) | 0 (0) | 2 (5) | 0.03 |
| In-hospital mortality | 19 (39) | 23 (18) | 2 (5) | 5 (12) | <0.001 |
| 28-day mortality | 13 (27) | 19 (15) | 2 (5) | 3 (7) | 0.02 |
| 90-day cumulative mortality^†^ | 19 (40) | 26 (21) | 3 (8) | 5 (12) | 0.001 |
| Use for KRT at day 28 | 4 (8) | 0 (0) | 0 (0) | 0 (0) | 0.001 |
| Length-of-ICU stay, median (IQR) | 12 (9–17) | 8 (5–12) | 8 (5–14) | 6 (5–12) | <0.001 |
| Length-of-hospital stay, median (IQR) | 29 (17–65) | 26 (17–47) | 40 (21–67) | 31 (22–57) | 0.19 |

^†^ Kaplan-Meier estimate showing the number of deaths (cumulative mortality). A total of 21 patients (8%) were lost to follow-up at 90 days.

Data are presented as median [interquartile range] or N (%). Variables were compared using Kruskal-Wallis test or chi-square test as appropriate.

This table presents a sensitivity analysis using an alternative definition of persistent severe SA-AKI—defined as a composite of (1) persistent KDIGO stage 3 AKI≥72 h, (2) dialysis, or (3) death following KDIGO stage 3 AKI. Transient severe SA-AKI was defined as patients with stage 2–3 AKI within 48 hours of ICU admission who did not meet the criteria for persistent severe SA-AKI.

Abbreviations: ICU, intensive care unit; IQR, interquartile range; KRT, kidney replacement therapy; SA-AKI, sepsis-associated acute kidney injury.

**Table S3. Association of persistent severe SA-AKI defined by an alternative definition with outcomes**

| **Primary outcome** | **Crude hazard ratio (95% CI)** | ***P* value** | **Adjusted hazard ratio (95% CI)** | ***P* value** |
| --- | --- | --- | --- | --- |
| 90-day mortality | 2.05 (1.14–3.71) | 0.02 | 2.24 (1.17–4.29) | 0.02 |
| **Secondary outcomes** | **Crude odds ratio (95% CI)** | ***P* value** | **Adjusted odds ratio (95% CI)** | ***P* value** |
| In-hospital mortality | 2.86 (1.38–5.97) | 0.01 | 3.20 (1.36–7.66) | 0.01 |

Cox proportional hazard model for 90-day mortality was fit, adjusted for age, sex, BMI, APACHE II score, and Charlson comorbidity index. Logistic regression models for in-hospital mortality and composite outcome at day 28 were fit, adjusted for age, sex, BMI, APACHE II score, and Charlson comorbidity index.

All comparisons were made between patients with persistent severe SA-AKI and those with transient severe SA-AKI, which served as the reference group. Persistent severe SA-AKI was defined as a composite of (1) persistent KDIGO stage 3 AKI≥72 h, (2) dialysis, or (3) death following KDIGO stage 3 AKI. Transient severe SA-AKI was defined as patients with stage 2–3 AKI within 48 hours of ICU admission who did not meet the criteria for persistent severe SA-AKI.

Abbreviations: APACHE, acute physiology and chronic health evaluation; BMI, body mass index; CI, confidence interval; KRT, kidney replacement therapy; SA-AKI, sepsis-associated acute kidney injury.

**Table S4. Association of persistent SA-AKI defined using a 72-hour threshold with outcomes**

| **Primary outcome** | **Crude hazard ratio (95% CI)** | ***P* value** | **Adjusted hazard ratio (95% CI)** | ***P* value** |
| --- | --- | --- | --- | --- |
| 90-day mortality | 3.48 (1.77–6.82) | <0.001 | 2.90 (1.43–5.87) | 0.003 |
| **Secondary outcomes** | **Crude odds ratio (95% CI)** | ***P* value** | **Adjusted odds ratio (95% CI)** | ***P* value** |
| In-hospital mortality | 4.36 (2.04–10.01) | <0.001 | 3.54 (1.52–8.76) | 0.004 |
| Composite outcome at day 28^†^ | 4.46 (1.96–11.15) | <0.001 | 3.13 (1.28–8.25) | 0.02 |

Cox proportional hazard model for 90-day mortality was fit, adjusted for age, sex, BMI, APACHE II score, and Charlson comorbidity index. Logistic regression models for in-hospital mortality and composite outcome at day 28 were fit, adjusted for age, sex, BMI, APACHE II score, and Charlson comorbidity index.

All comparisons were made between patients with persistent SA-AKI and those with transient SA-AKI, which served as the reference group. Persistent SA-AKI was defined using a 72-hour threshold. Among patients with early SA-AKI, 92 patients had persistent SA-AKI, and 115 patients had transient SA-AKI. 8 patients were excluded from this analysis because they died before the determination of trajectory of SA-AKI.

Abbreviations: APACHE, acute physiology and chronic health evaluation; BMI, body mass index; CI, confidence interval; CKD, chronic kidney disease; KRT, kidney replacement therapy; SA-AKI, sepsis-associated acute kidney injury.

**Table S5. Association of persistent SA-AKI with outcomes in patients without pre-existing CKD**

| **Primary outcome** | **Crude hazard ratio (95% CI)** | ***P* value** | **Adjusted hazard ratio (95% CI)** | ***P* value** |
| --- | --- | --- | --- | --- |
| 90-day mortality | 3.38 (1.76–6.50) | <0.001 | 2.70 (1.36–5.35) | 0.004 |
| **Secondary outcomes** | **Crude odds ratio (95% CI)** | ***P* value** | **Adjusted odds ratio (95% CI)** | ***P* value** |
| In-hospital mortality | 4.97 (2.33–11.61) | <0.001 | 4.22 (1.83–10.56) | 0.001 |
| Composite outcome at day 28^†^ | 5.48 (2.41–14.17) | <0.001 | 4.01 (1.66–10.85) | 0.003 |

Cox proportional hazard model for 90-day mortality was fit, adjusted for age, sex, BMI, APACHE II score, and Charlson comorbidity index. Logistic regression models for in-hospital mortality and composite outcome at day 28 were fit, adjusted for age, sex, BMI, APACHE II score, and Charlson comorbidity index.

All comparisons were conducted between patients with persistent SA-AKI and those with transient SA-AKI, after excluding patients with CKD. The transient SA-AKI group served as the reference.

Abbreviations: APACHE, acute physiology and chronic health evaluation; BMI, body mass index; CI, confidence interval; CKD, chronic kidney disease; KRT, kidney replacement therapy; SA-AKI, sepsis-associated acute kidney injury.

**Table S6. Outcomes of patients with septic shock stratified by the pattern of SA-AKI using the different criteria for estimating baseline creatinine level**

|  | **Total (N=257)** | **Persistent SA-AKI (N=111)** | **Transient SA-AKI (N=105)** | **No SA-AKI (N=41)** | **P value** |
| --- | --- | --- | --- | --- | --- |
| ICU mortality | 26 (10) | 20 (18) | 4 (4) | 2 (5) | 0.001 |
| In-hospital mortality | 49 (19) | 35 (32) | 9 (9) | 5 (12) | <0.001 |
| 28-day mortality | 37 (14) | 27 (24) | 7 (7) | 3 (7) | <0.001 |
| 90-day cumulative mortality^†^ | 53 (21) | 36 (32) | 12 (11) | 5 (12) | <0.001 |
| Use for KRT at day 28 | 4 (2) | 4 (4) | 0 (0) | 0 (0) | 0.07 |
| Length-of-ICU stay, median (IQR) | 8 (5–14) | 9 (6–14) | 8 (5–13) | 6 (5–12) | 0.17 |
| Length-of-hospital stay, median (IQR) | 28 (18–60) | 26 (17–60) | 30 (19–64) | 30 (22–52) | 0.54 |

^†^ Kaplan-Meier estimate showing the number of deaths (cumulative mortality). A total of 21 patients (8%) were lost to follow-up at 90 days.

Data are presented as median [interquartile range] or N (%). Variables were compared using Kruskal-Wallis test or chi-square test as appropriate.

Abbreviations: ICU, intensive care unit; IQR, interquartile range; KRT, kidney replacement therapy; SA-AKI, sepsis-associated acute kidney injury.

**Table S7. Association of persistent SA-AKI with outcomes in patients receiving noradrenaline >0.4 μg/kg/min**

| **Primary outcome** | **Crude hazard ratio (95% CI)** | ***P* value** | **Adjusted hazard ratio (95% CI)** | ***P* value** |
| --- | --- | --- | --- | --- |
| 90-day mortality | 2.88 (1.06–7.83) | 0.04 | 2.54 (0.89–7.27) | 0.08 |
| **Secondary outcomes** | **Crude odds ratio (95% CI)** | ***P* value** | **Adjusted odds ratio (95% CI)** | ***P* value** |
| In-hospital mortality | 7.41 (1.98–36.81) | 0.006 | 8.11 (1.82–49.70) | 0.01 |
| Composite outcome at day 28^†^ | 4.33 (1.15–21.45) | 0.04 | 3.82 (0.87–21.64) | 0.09 |

The analysis was restricted to 50 patients receiving noradrenaline at a dose >0.4 μg/kg/min.

Cox proportional hazard model for 90-day mortality was fit, adjusted for age, sex, BMI, APACHE II score, and Charlson comorbidity index. Logistic regression models for in-hospital mortality and composite outcome at day 28 were fit, adjusted for age, sex, BMI, APACHE II score, and Charlson comorbidity index.

All comparisons were conducted between patients with persistent SA-AKI and those with transient SA-AKI. The transient SA-AKI group served as the reference.

Abbreviations: APACHE, acute physiology and chronic health evaluation; BMI, body mass index; CI, confidence interval; CKD, chronic kidney disease; KRT, kidney replacement therapy; SA-AKI, sepsis-associated acute kidney injury.

**Table S8. Adjusted associations of factors related to hemodynamic status and management with the development of persistent SA-AKI in different models in sensitivity analysis**

| Variable | Univariable OR (95% CI) | *P* value | Multivariable OR (95% CI) | *P* value |
| --- | --- | --- | --- | --- |
| TWA MAP during the first 48 hours | 0.98 (0.95–1.01) | 0.21 | 1.00 (0.96–1.04) | 0.94 |
| TWA VIS during the first 48 hours | 1.04 (1.02–1.06) | 0.001 | 1.03 (1.01–1.06) | 0.04 |
| Cumulative fluid balance by day 3, per 100 ml | 1.01 (1.00–1.01) | 0.04 | 1.01 (1.00–1.01) | 0.16 |
| Maximum serum lactate level on day 1 | 1.10 (1.02–1.20) | 0.02 | 1.04 (0.95–1.14) | 0.42 |
| Age | 1.00 (0.98–1.03) | 0.71 | 1.01 (0.98–1.04) | 0.52 |
| Male sex | 1.17 (0.68–2.00) | 0.57 | 0.87 (0.46–1.63) | 0.66 |
| BMI | 1.08 (1.02–1.14) | 0.01 | 1.09 (1.02–1.16) | 0.02 |
| CCI | 1.12 (0.94–1.34) | 0.20 | 1.02 (0.83–1.25) | 0.87 |
| APACHE II score | 1.10 (1.06–1.15) | <0.001 | 1.08 (1.03–1.14) | 0.002 |
| MV within the first 6 hours of ICU admission | 1.51 (0.83–2.76) | 0.17 | 1.05 (0.51–2.14) | 0.89 |
| Source of infection (urinary tract/abdomen or not) | 1.05 (0.61–1.79) | 0.87 | 1.13 (0.59–2.20) | 0.71 |
| Blood culture positivity | 1.77 (1.03–3.07) | 0.04 | 1.43 (0.75–2.71) | 0.28 |

A multivariable logistic regression model was fitted to evaluate the association of hemodynamic status and management-related factors—including MAP, VIS, cumulative fluid balance, and serum lactate level—with the development of persistent SA-AKI. TWA MAP and VIS were calculated using 2-hour interval data from ICU admission to 48 hours, with each measurement weighted by the duration between successive time points. The model was adjusted for age, sex, BMI, CCI, APACHE II score, use of MV within the first 6 hours of ICU admission, source of infection (urinary tract/abdomen or not), and blood culture positivity.

Abbreviations: APACHE, acute physiology and chronic health evaluation; BMI, body mass index; CCI, Charlson Comorbidity Index; ICU, intensive care unit; MAP, mean arterial pressure; MV, mechanical ventilation; SA-AKI, sepsis-associated acute kidney injury; TWA, time-weighted average; VIS, vasoactive-inotropic score.

**Figure S1. Patient flow**


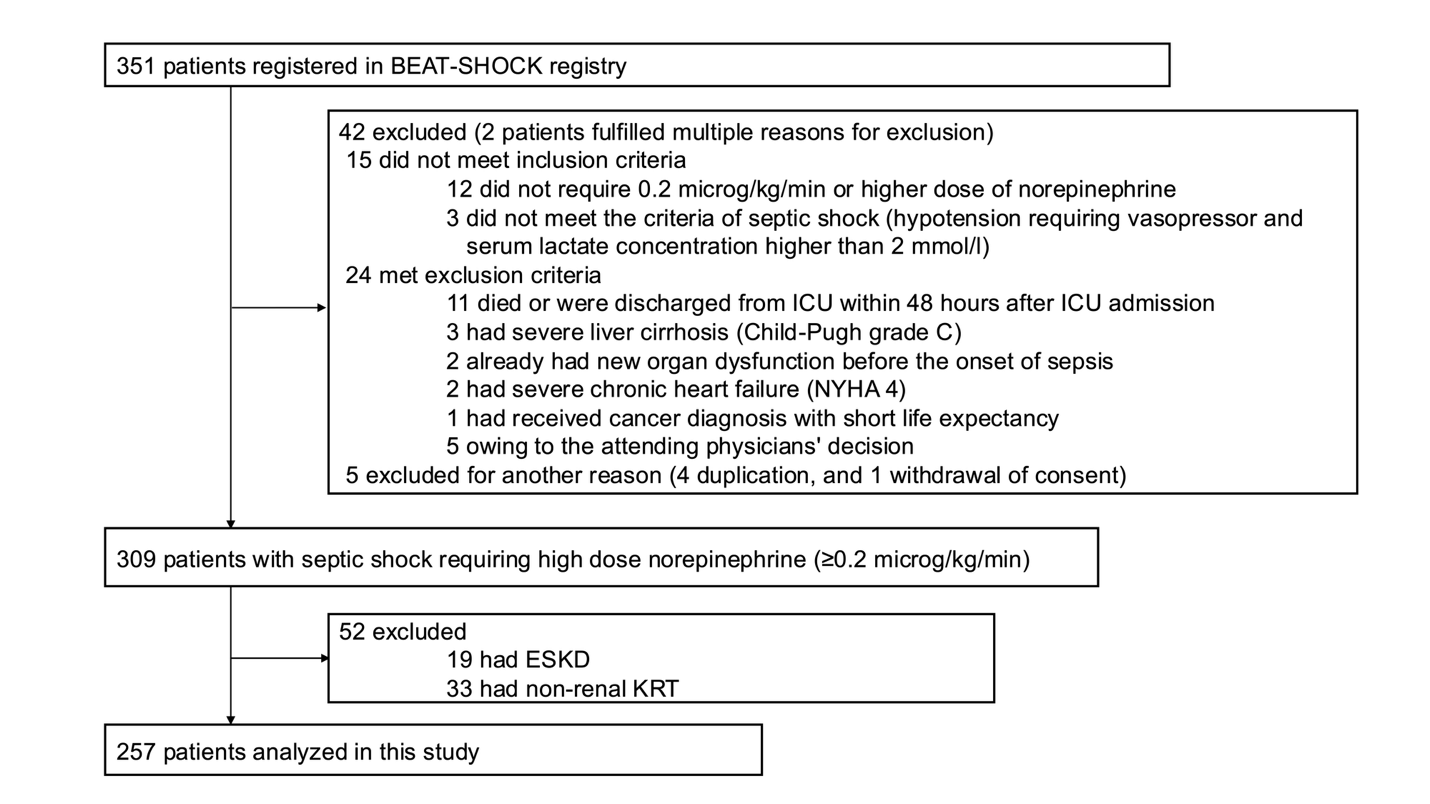


A total of 351 patients were registered in BEAT-SHOCK registry. After excluding 42 patients, 309 patients with septic shock requiring high-dose norepinephrine were included in the registry. After excluding 52 patients who had ESKD or non-renal KRT, 257 patients were analyzed in this analysis.

Abbreviations: ESKD, end-stage kidney disease; ICU, intensive care unit; NYHA, New York Heart Association; KRT, kidney replacement therapy.
